# Are Dietitians With Obesity Perceived as Competent and Warm? Applying the Stereotype Content Model to Weight Stigma in Brazil

**DOI:** 10.3389/fnut.2022.813344

**Published:** 2022-02-25

**Authors:** Giovana Santarosa Cassiano, Joana Pereira Carvalho-Ferreira, Nicola J. Buckland, Mariana Dimitrov Ulian, Diogo Thimoteo da Cunha

**Affiliations:** ^1^Multidisciplinary Food and Health Laboratory, School of Applied Sciences, State University of Campinas, Campinas, Brazil; ^2^Department of Psychology, University of Sheffield, Sheffield, United Kingdom; ^3^Department of Nutrition, School of Public Health, University of São Paulo, São Paulo, Brazil

**Keywords:** obesity, Stereotype Content Model (SCM), antifat attitudes, weight stigma, weight bias, counseling

## Abstract

The aim of this study was to understand how dietitians' body size influences perceived competence and warmth, based on the Stereotype Content Model (SCM). Online data were collected from 1,039 Brazilians, who were either laypeople, registered dietitians, or nutrition students. Participants rated the competence and warmth dimensions of three dietitians who differed in sex, body weight, and age. Participants also indicated how likelythey would consult or recommend each dietitian for nutritional advice, and indicated their attitudes toward people with obesity (PWO) [using The Antifat Attitudes Test (AFAT)]. Laypeople attributed less competence and warmth to all profiles compared to dietitians and students (*p* < 0.001). Three clusters occupied the SCM warmth-by-competence space. However, the clusters were different among groups (laypeople, dietitians, and students). For lay participants, the woman without overweight, the older woman, and the older man were located in the high competence/medium warmth cluster. Meanwhile, the woman with obesity was located in the medium competence/high warmth cluster. The dietitians and students map found the woman with obesity and the older woman in a high competence and warmth cluster. In general, the woman with obesity, the man without obesity, and the older man can be classified as ambivalent stereotypes, the woman being perceived as more warm than competent and the men more competent than warm. Participants with high AFAT scores were less likely to consult or recommend to a family member a dietitian with obesity. This study contributes to identifying ambivalent stereotypes for dietitians. Dietitians with obesity can be seen as warm but less competent. Also, although less intense than laypeople, dietitians, and students exhibited weight stigma. These findings can foster important discussions about weight stigma and emphasize the need to increase population awareness about the causes of obesity.

## Introduction

Stereotypes are a set idea that people or groups share about what someone or something is like (1). This idea is, generally, a wrong or stigmatized view about the object or person. However, according to the Stereotype Content Model (SCM) (2), stereotypes are not just negative; many societal stereotypes are ambivalent, combining a group's positive and negative characteristics. Based on this model, stereotypes array along two fundamental dimensions of social perception, namely, warmth (sociability, sincerity) and competence (capability, skill) (3). Ambivalent stereotypes portray groups as either warm but not competent for historically perceived compliant subordinates (e.g., disabled people, women) or as cold but competent for successful competitors (e.g., rich people). Groups penalized on one dimension are compensated on the other (4). The positive description may mask the negative description, making ambivalent stereotypes acceptable even to targets (5). Mixed combinations rationalize the system. This ambivalent (or mixed) stereotype promotes existing systems of privilege and placates the non-threatening but disadvantaged out-groups by assigning them socially desirable, though subordinating, traits (6). Socioeconomically successful out-groups, however, pose a competitive threat, and their success elicits envy. For envied out-groups, the mixed stereotype explains their apparent success, thereby justifying the system of meritocracy that benefits societal reference groups and dominant in-groups. Stereotypes of low warmth justify taking action against envied groups by casting the groups as being concerned only with furthering their own goals. Thus, envied groups may be appropriately resented and socially excluded (2). Fiske and collaborators's (2), data from nine survey samples supported their hypotheses regarding stereotype content. The cluster analyses, conducted with various group selection methods, found evidence for the dimensional hypothesis that perceived competence and warmth differentiate out-group stereotypes. These studies also supported the mixed stereotypes hypothesis that many out-groups are viewed as competent but not warm or viewed as not competent but warm. They also found social structural correlates of perceived competence and warmth. That is, perceived social status predicted perceived competence, whereas perceived competition predicted perceived lack of warmth. Finally, the authors addressed the emotional concomitants of different stereotype contents, showing that pity (low competence and high warmth), envy (high competence with low warmth), contempt (low competence and warmth), and admiration (with competence and warmth) differentiated the four combinations of perceived warmth and competence (2).

Socioculturally, individuals with overweight or obesity, are associated with being happier and more emotional than people without obesity—known as the “fat-jolly” hypothesis in sociology and social psychology (7, 8). A study demonstrated that people with overweight are evaluated as warmer than people without obesity because a large body size signals greater emotional expressiveness (9). Similarly, warmth is attributed to marketers who display broad, intense smiles (i.e., indicating a high intensity of emotion) (10), and emotionality in decision-making signals a willingness to cooperate—a warm intention (11). Regarding competence, people without obesity are perceived to exercise self-control than people with overweight (12, 13). This is taken as a necessary characteristic to become skilled and effective at any task, as the person must devote time and effort to the task, exert willpower to stay focused, and delay the enjoyment of pleasure (9). So, a “normal weight” signals the ability to exercise self-control, whereas heaviness signals a lack of this quality (9). Levine and Schweitzer (14) also demonstrated that obesity is intimately linked with perceptions of low competence and these perceived assumptions reflect bias. This illustrates how people with obesity (PWO) can be stereotyped, affecting their personal life and work (15, 16).

People with heavier body weight are targets of weight stigma, being stereotyped as lazy, weak-willed, unsuccessful, unintelligent, lacking self-discipline, and having little willpower (15, 17). These are moral judgments that discredit people with higher body weight (18). Goffman (19) describes stigma as a deeply discrediting attribute, disqualifying a person from full social acceptance. Having a heavier bodyweight is currently socially understood as a “deviant” characteristic, which distances someone from those who are supposedly “normal” [i.e., classified with normal weight according to the World Health Organization (20)] and this stigmatizing feature legitimizes a series of social discriminations, slanders, or exclusions. Weight stigma can hinder the possibility of PWO to care for their health. Studies have shown that this population avoids health care services because they are concerned about being stigmatized due to their weight (21). Health professionals can also be a target of stigmas (22–24). For dietitians with obesity, their weight can lead to stigmatized comments and attitudes. In public perception, a dietitian with overweight or obesity represents an inversion of the normative values of nutrition, leading to the disapproval of dietitians with obesity (25). Because dietitians have specific knowledge of nutrition and treat PWO by advising lifestyle changes, dietitians may be expected to have a body with “adequate weight.” Therefore, dietitians with overweight may be seen as less competent in their professional performance than those without overweight or obesity (25). To date, the SCM has not been explicitly applied to assess the relationship between obesity and dietitians. This study seeks to advance the evidence of public perceptions of dietitians with obesity, and the consequences of weight stigma for dietitians' professional practice. In line with SCM, we hypothesize: (A) that dietitians with different profiles—varying in body weight, age, and gender—may be associated with ambivalent stereotypes; (B) that profiles with obesity may be evaluated as less competent, decreasing participants' likelihood of consulting or recommending them.

The study's first objective was to draw on the framework of competence and warmth dimensions, based on the SCM, to assess how dietitians' body size influences manifestations of weight stigma made by the general public (laypeople), and peers—i.e., registered dietitians and nutrition students. The second objective was to assess whether weight status affects the likelihood of a dietitian being consulted or recommended based on participants' antifat attitudes.

## Methods

### Sample, Design, and Data Collection

All data were collected using the online platform Google Forms (Alphabeth Inc. Mountain View, CA, USA) from July to December 2020. Only Brazilians 18 years and older were enrolled. A non-probability purposive, with chain-referral sampling, was employed. Due to the COVID-19 pandemic, all participants were recruited online through social media. The sample comprised of three different groups: laypeople, registered dietitians, and nutrition students. The minimum sample was established considering three groups, effect size *f* = 0.15, alpha error of 5%, and sample power of 90%. The effect size was specified based on a pilot test with 152 volunteers (this data was not included in this study). Calculations showed a minimum of 188 participants were required in each group. Since the study population is accessible, large, and online research was employed, an increased sample number of *n* > 300 was defined for laypeople and *n* > 250 for each dietitian and nutrition student to reduce sampling error and increase heterogeneity. The standard deviation (SD) between the indicator variables (warmth and competence dimensions) was checked for each participant. Participants with SD = 0 were excluded from being indicative of poor data. The study's true purpose was concealed from participants to minimize the risk of bias. The title of the online survey was “Evaluation of dietitians' work and the factors related to obesity.” The study was conducted according to the guidelines of the Declaration of Helsinki and approved by the University of Campinas Ethics Committee (Protocol: 30637020.5.0000.5404 approved on 5 June 2020). Participants signed an informed consent and did not receive any incentives for participation, in line with Brazilian ethics regulation (26).

For this study, we classified nutritional status based on the use of “person first” (e.g., PWO) (27).

### Manipulating the Weight Status of Dietitians

**Figure 1:**
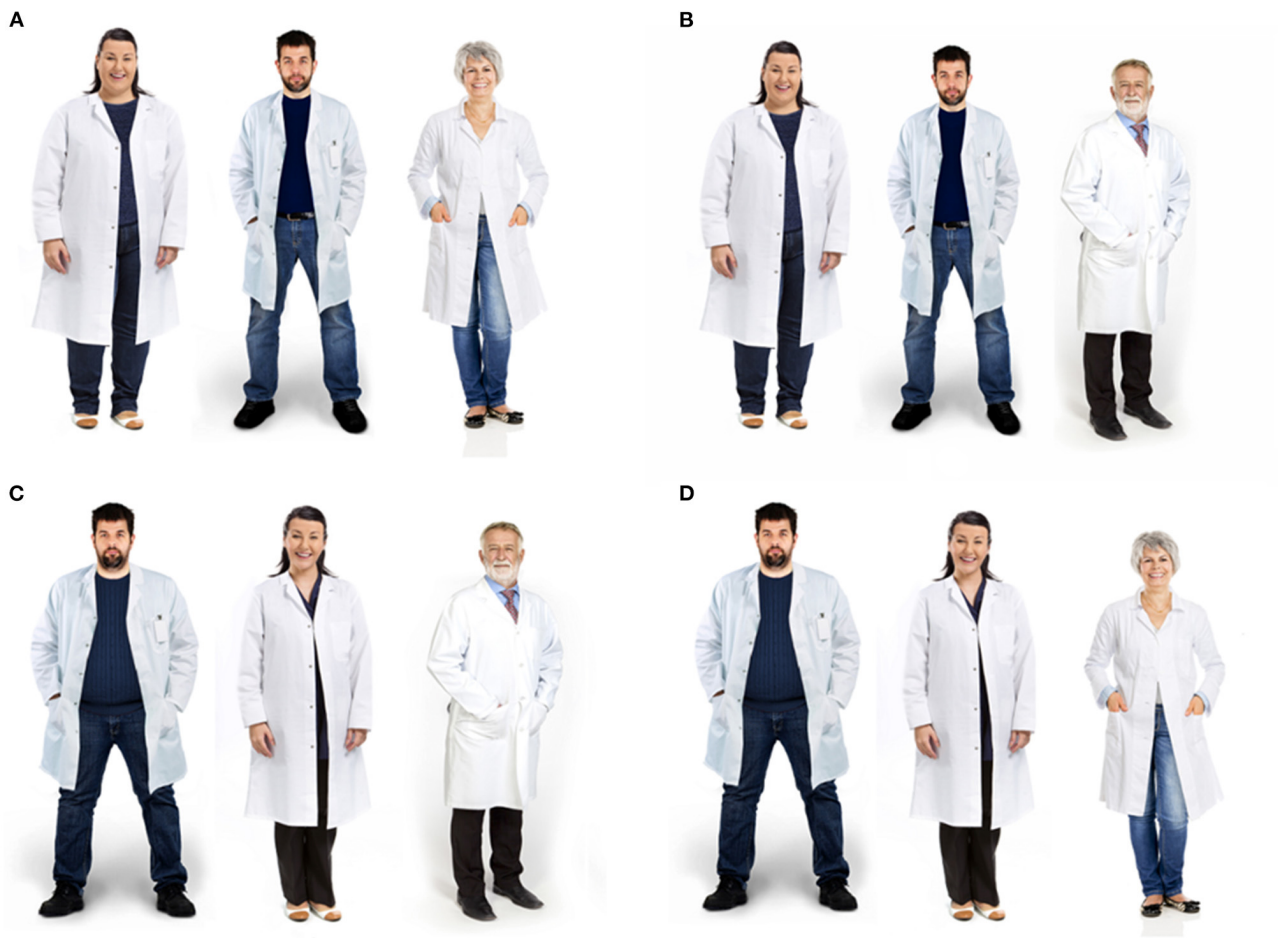
Dietitians varying in weight, gender, and age (presented in different blocks). **(A)** Woman with obesity, man without obesity, older woman; **(B)** Woman with obesity, man without obesity, older man; **(C)** Man with obesity, woman without obesity, older man; **(D)** Man with obesity, woman without obesity, older woman.

Each participant evaluated one block (A, B, C, or D) of three profiles of dietitians, as shown in Figure 1. A complete randomized block design was used. This procedure was chosen to avoid an evaluation bias by identifying the same person with two different weights, biasing their response. Each block has a model with obesity (male or female), and two models without obesity, younger and older, from both sexes. Images were purchased from an image bank. It was included a photo from the same model pre- and post-bariatric surgery for younger models. This procedure has already been performed in other research that addressed weight stigma (28) due to the difficulty of finding matching images in terms of attractiveness, dress pattern, age, and ethnicity. This approach was also used due to failed attempts by computer morphing techniques to create average weight figures from obese target photos and vice versa (28). Using pre- and post-bariatric surgery images, it is possible to compare ratings for the same target but in different weight statuses (with obesity vs. without obesity), overcoming the difficulties of combining different targets (29). The older man and woman were included as control, since they are known high-warmth stereotypes (3). The images were manipulated in Photoshop software by a professional in which the white coat was included, and minor aesthetic changes were made to approximate the images. To validate the images, an online pilot study with 61 laypeople rated the images considering age, weight (considering 1.70 m tall for men and 1.60 m for women), and sex. The panel rated profiles with obesity to have a greater body weight compared to profiles without obesity (*p* < 0.001). Older profiles were rated as significantly older compared to the younger profiles (*p* < 0.001). Participants were also able to distinguish the sex of the profiles (100% accuracy). A full description of the validation is included as a Supplementary Material.

### Measures

In line with SCM, each profile was evaluated for warmth and competence (2). An abbreviated and adapted version of the SCM questionnaire was used, in which six items represented competence and warmth traits (three for each dimension) (Table 1). Responses were given for each profile one at a time. Each item was answered on a five-point Likert-type scale “1—Not at all” to “5—Extremely.” In addition, nutrition students and dietitians answered the following questions: “Would you recommend this dietitian to a friend or family member?” Answering yes or no. Lay participants answered: “If you needed nutritional advice, would you consult with this dietitian?” answering yes or no. All measures were administered in Brazilian Portuguese.

**Table 1:** Items in the abbreviated “SCM” questionnaire assessing competence and warmth.

**Construct**	**Items**
Competence	• Analyzing only the image of this dietitian, how… 1. Competent do you believe she/he is? 2. Confident do you believe she/he is? 3. Intelligent do you believe she/he is?
Warmth	• Analyzing only the image of this dietitian, how… 1. Sincere do you believe she/he is? 2. Good-natured do you believe she/he is? 3. Warm do you believe she/he is?

Antifat attitudes of all participants were assessed using the The Antifat Attitudes Test (AFAT) (30, 31). The AFAT consists of 34 items, divided into three subscales: Factor 1—Social and character depreciation (15 items), ascribing socially undesirable personality characteristics and social disregard for PWO; Factor 2—Physical/romantic unattractiveness (10 items), reflecting perceptions that PWO are clumsy, unattractive, and are unacceptable as romantic partners, and; Factor 3—Weight control/blame (nine items) that assesses beliefs concerning whether PWO are responsible for their weight. A global score was calculated considering all items' means. All the scales presented adequate composite reliability: factor 1 = 0.78, factor 2 = 0.84, factor 3 = 0.84, global score = 0.72. The detailed results of AFAT are discussed elsewhere (32). To not bias the answers, participants answered the AFAT after answering the other questionnaires. This step was performed to assess whether negative attitudes toward PWO would influence the ratings assigned to the dietitians.

Finally, a questionnaire was developed whereby participants indicated all the adjectives (check-all-that-apply) that can be attributed to a “good dietitian.” The term “good dietitian” was used to avoid using adjectives similar to the SCM evaluation, such as competent, intelligent, sincere, etc. A list with 28 adjectives—informed from pilot work—such as communicative, innovative, within the ideal weight, attentive, among others, was presented to each participant. The selected adjectives comprised six groups: physical appearance, professional appearance (dress code), warmth-oriented, competence-oriented, and social- and innovativeness-oriented. Participants were instructed to select all that apply. Although some of the adjectives used are stigmatizing, they were used intentionally because they are adjectives commonly used in communication, magazines, and social networks.

### Analysis

All variables were analyzed for distribution by mean analysis, SD, histograms, kurtosis, skewness, and normality by the Kolmogorov-Smirnov test with Lilliefors correction homoscedasticity by Levene's test. No differences were found between blocks, so this variable was not included in the analysis as control. Stereotype-related ratings were compared using Analysis of Variance (ANOVA) for repeated measures with Greenhouse-Geisse correction. Bonferroni *post-hoc* for multiple comparisons was used. Pearson correlations were used to assess the relationship between attitudes toward PWO and the scores of competence and warmth in the profiles of dietitians. One-way ANOVA with Welch correction was used to compare three groups (laypeople, dietitians, and students). The omega-squared (ω^2^) was used to measure the effect size of ANOVA statistics. The effect size (ω^2^) was classified as small (ω^2^ = 0.01), medium (ω^2^ = 0.06), and large (ω^2^= 0.14) (33). The composite reliability higher than 0.70 was used to ascertain dimensions/factor's reliability.

In line with Cuddy et al. (34), a two cluster procedure was conducted following the SCM. First, hierarchical cluster analysis with Ward's method considering competence and warmth dimensions of each profile was conducted. The interval measure was set to Squared Euclidian Distance. Based on the elbow chart and ANOVA, a three-cluster solution was selected. After, a k-means cluster analysis using the parallel threshold method to reveal the cluster memberships (35). The results were plotted in an SCM map for each group (laypeople, dietitians, and students).

Student's *t*-test for paired samples was used to compare two related samples. Student's *t*-test was used for two independent groups. Cohen's *d* was used to verify the effect size of *t*-statistics. The effect size (*d*) was classified as small (*d* = 0.2), medium (*d* = 0.5), and large (*d* = 0.8) (36). The stereotype ambivalence was defined when competence and warmth dimensions scores, within the same stereotype, have differences (*p* < 0.05) with at least medium effect size (*d* > 0.50) (35). The AFAT scores were normalized using log-transformation. The Bonferroni's correction (0.05/3 = 0.0125) avoided family-wise error when assessing the AFAT scores between two groups. For all other analyses, a *p*-value of <0.05 was considered significant. Statistical analyses were performed using Statistical Package for Social Sciences (SPSS) v.20 (IMB) and JASP 0.14.1 (University of Amsterdam).

## Results

### Sample Characteristics

The total sample consisted of 1,051 individuals. Twelve individuals were excluded since their responses showed no variance (SD = 0). The final sample was 1,039 individuals, separated into laypeople (*n* = 403), dietitians (*n* = 336), and nutrition students (*n* = 300). Laypeople were mainly female (64%), with an average (SD) age of 32.0 (11.93) years, and with a BMI of 26.29 (6.34) kg/m^2^ (range = 15.2–44.9 kg/m^2^). Most were engaged in (33.7%) or had completed (25.3%) higher education, with family income higher than five Brazilian minimum wages (41%). Dietitians were primarily female (94.6%), aged 32.54 (9.12) years old and BMI of 24.72 (4.21) kg/m^2^ (range = 16.3–40.6 kg/m^2^). Most were postgraduate (56.8%) with family income higher than five Brazilian minimum wages (46.7%). Nutrition students were also primarily female (92.7%), with a mean age of 25.12 (7.20) years and BMI of 24.04 (4.33) kg/m^2^ (range = 16.0–40.7 kg/m^2^). Most nutrition students had a family income between two and five Brazilian minimum wages (37.7%). Laypeople had a significantly higher BMI compared to dietitians and students (*p* < 0.05). Students were significantly younger than dietitians and laypeople (*p* < 0.05). The proportion of females was higher in the dietitians and students group than laypeople (*p* < 0.05).

### SCM Evaluation

**Figure 2:**
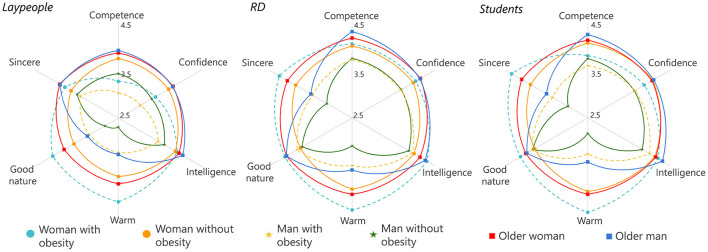
SCM evaluation in laypeople, registered dietitians (RD), and nutrition students.

The details about the SCM evaluation, including means, SDs, and internal consistency, can be found in Supplementary Materials. Competence and warmth scales showed satisfactory reliability for each profile, with composite reliability ranging from 0.74 to 0.95. As depicted in Figure 2, laypeople attributed less competence and warmth for all profiles than dietitians and students (*p* < 0.001). The effect size was larger when evaluating the competence of the profiles with obesity—Man: ω^2^ = 0.12; Women: ω^2^ = 0.10 with medium effect size. For other profiles, the differences between laypeople and dietitians/students for competence and warmth presented small effects η^2^ < 0.05. No differences were found between dietitians and students. Still, all three groups rated the man without obesity and the man with obesity more negatively on both dimensions. No correlation was found between participants' BMI and competence and warmth for all profiles (*r* < 0.15).

**Figure 3:**
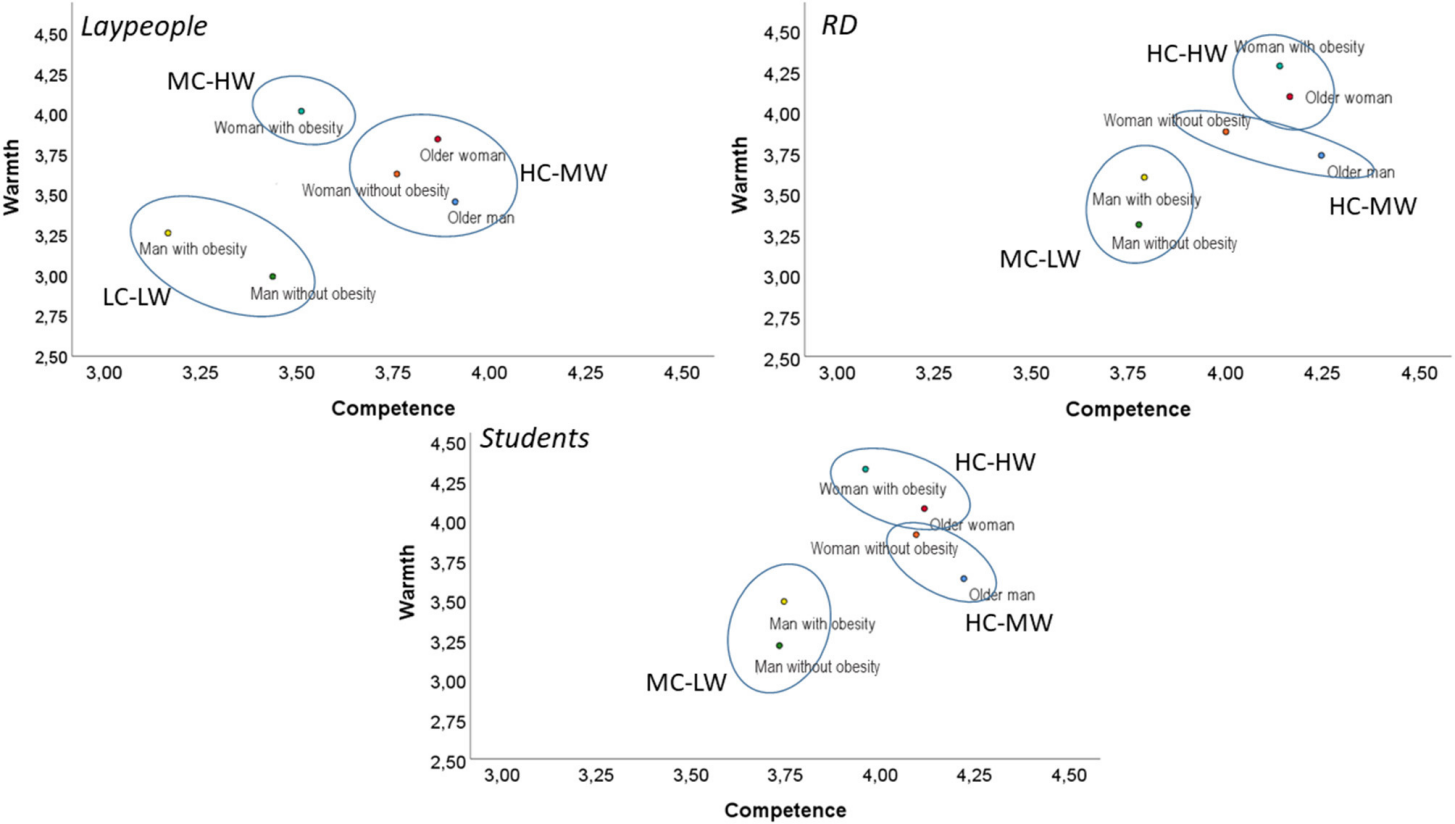
The Stereotype Content Model (SCM) map of dietitians stereotypes. *MC-HW, Middle Competence-High Warmth; HC-MW, High Competence-Middle Warmth; LC-LW, Low Competence-Low Warmth; HC-HW, High Competence-High Warmth; MC-MW, Middle Competence-Middle Warmth; MC-LW, Middle Competence-Low Warmth.

The hierarchical cluster revealed agglomeration statistics that supported a three-cluster solution, grouped into three quadrants in the SCM map (Figure 3). We found five clusters: high competence and high warmth (HC-HW), high competence and middle warmth (HC-MW), middle competence and high warmth (MC-HW), middle competence and low warmth (MC-LW), and low competence and low warmth (LC-LW). High competence and low warmth (HC-LW) and low competence and high warmth (LC-HW) cluster did not appear. The paired sample *t*-test comparisons of competence and warmth mean for each cluster appear in Table 2. The clusters were named based on the scores and analysis presented in Table 2 and SCM map (Figure 3).

**Table 2:** Competence and warmth means for each cluster.

**Group**	**Cluster**	**Competence**		**Warmth**	p^*^	***d***
		**Mean (SD)**		**Mean (SD)**		
Laypeople	MC-HW	3.51 (0.79)	<	4.02 (0.68)	<0.001	0.72
	HC-MW	3.84 (0.68)	>	3.65 (0.68)	<0.001	0.32
	LC-LW	3.30 (0.75)	>	3.13 (0.74)	<0.001	0.25
Registered	HC-HW	4.15 (0.67)	=	4.18 (0.63)	0.23	0.06
Dietitians	HC-MW	4.12 (0.71)	>	3.80 (0.75)	<0.001	0.52
	MC-LW	3.78 (0.78)	>	3.45 (0.85)	<0.001	0.48
Students	HC-HW	4.03 (0.79)	<	4.20 (0.66)	<0.001	0.24
	HC-MW	4.16 (0.64)	>	3.76 (0.70)	<0.001	0.31
	MC-LW	3.74 (0.76)	>	3.34 (0.80)	<0.001	0.32

*M, mean; SD, standard deviation; MC-HW, Middle Competence-High Warmth; HC-MW, High Competence-Middle Warmth; LC-LW, Low-Competence-Low Warmth; HC-HW, High Competence-High Warmth; HC-MW, High Competence-Middle Warmth; MC-LW, Middle Competence-Low Warmth*.

* *Paired Student's t-test*.

In Figure 3, it is possible to see that three cluster solutions for each evaluated group (laypeople, dietitians, and nutrition students) were different. In the laypeople group, the woman without obesity, the older woman, and the older man was located in the HC-MW cluster. Meanwhile, the woman with obesity was located in the MC-HW cluster. Both man with obesity and without it were located in the LC-LW cluster—being lower in warmth and competence with a small effect size (*d* = 0.25). Dietitians and nutrition students' SCM maps were similar but differed from the laypeople's evaluation. In both groups (dietitians and students), the woman with obesity and the older woman were located in the HC-HW cluster. Similarly, the woman without obesityand the older man were located in the HC-MW cluster. On the other hand, they perceived the man without obesityand the man with obesity with a middle competence and lower warmth (MC-LW cluster).

### Ambivalent Stereotypes

In general, three profiles can be classified as ambivalent sterotypes: the woman with obesity, the man without obesity, and the older man (see Supplementary Tables for details). The woman with obesity was rated significantly more warm than competent. The men's profiles were rated significantly more competent than warm. Some differences were observed among the studied groups. Laypeople rated the woman with obesity significantly more warm than competent (*M*_diff_ = 0.50, *p* < 0.001, *d* = 0.71), the man without obesity more competent than warm (*M*_diff_ = 0.44, *p* < 0.001, *d* = 0.73) as well as the older man (*M*_diff_ = 0.46, *p* < 0.001, *d* = 0.72), all of the three with medium effect size. Dietitians rated the man without obesity more competent than warm (*M*_diff_ = 0.47, *p* < 0.001, *d* = 0.75), as well as the older man (*M*_diff_ = 0.50, *p* < 0.001, *d* = 0.79) with medium effect size. Finally, nutrition students rated the woman with obesity more warm than competent with medium effect size (*M*_diff_ = 0.36, *p* < 0.001, *d* = 0.52), the man without obesity (*M*_diff_ = 0.52, *p* < 0.001, *d* = 0.85), and the older man (*M*_diff_ = 0.60, *p* < 0.001, *d* = 0.93) more competent than warm, the last two with large effect size.

### Recommending Dietitian and Antifat Attitudes

Tables 3, 4 shows the percentage of participants who would consult and recommend each dietitian, respectively. In the laypeople group, the percentage of participants who would consult the dietitians with obesity was lower than the percentage of participants who would consult the dietitian with a normal weight. Compared to participants who would consult the dietitian, participants who would not consult the dietitian showed higher AFAT scores for subscales 2 and 3 and the AFAT global score, with medium effect size (*p* < 0.001, *d* ranging from 0.35 to 0.62) (Table 3). On the other hand, 92.4% would consult the woman without obesity, 77.5% the man without obesity, 92.4% the older woman, and 90.0% the older man, with no difference between the “Yes” or “No” answers and the AFAT scores.

**Table 3:** Laypeople's willingness to consult each dietitian profile and Antifat Attitudes Test (AFAT) scales.

	**If you need nutritional advice, would you consult with this dietitian?**				
**Profiles (% yes answers)**	**Variables**	**Yes**	**No**	***p*** ^*^	**d**
Woman with obesity	AFAT—Factor 1	1.25 (0.28)	1.35 (0.34)	0.032	0.36
(76.5%)	AFAT—Factor 2	1.67 (0.47)	1.95 (0.49)	**<0.001**	**0.57**
	AFAT—Factor 3	1.96 (0.61)	2.35 (0.69)	**<0.001**	**0.60**
	AFAT—Global score	1.56 (0.36)	1.79 (0.39)	**<0.001**	**0.62**
Woman without obesity	AFAT—Factor 1	1.27 (0.30)	1.30 (0.26)	0.70	0.13
(92.4%)	AFAT—Factor 2	1.73 (0.50)	1.73 (0.40)	0.97	0.04
	AFAT—Factor 3	2.10 (0.70)	2.30 (0.72)	0.29	0.28
	AFAT—Global score	1.62 (0.40)	1.69 (0.33)	0.53	0.20
Man with obesity	AFAT—Factor 1	1.27 (0.31)	1.29 (0.28)	0.56	0.08
(65.4%)	AFAT—Factor 2	1.67 (0.49)	1.83 (0.48)	**0.011**	**0.35**
	AFAT—Factor 3	2.02 (0.71)	2.30 (0.65)	**0.003**	**0.45**
	AFAT—Global score	1.58 (0.41)	1.71 (0.35)	**0.011**	**0.38**
Man without obesity	AFAT—Factor 1	1.27 (0.31)	1.27 (0.26)	0.92	0.01
(77.5%)	AFAT—Factor 2	1.74 (0.47)	1.72 (0.57)	0.48	0.12
	AFAT—Factor 3	2.03 (0.64)	2.12 (0.66)	0.37	0.15
	AFAT—Global score	1.61 (0.38)	1.63 (0.40)	0.85	0.03
Older woman	AFAT—Factor 1	1.27 (0.31)	1.40 (0.25)	0.12	0.44
(92.4%)	AFAT—Factor 2	1.74 (0.48)	1.91 (0.53)	0.30	0.29
	AFAT—Factor 3	2.08 (0.66)	2.48 (0.67)	0.03	0.59
	AFAT—Global score	1.62 (0.39)	1.82 (0.39)	0.07	0.52
Older man	AFAT—Factor 1	1.27 (0.29)	1.26 (0.30)	0.90	0.02
(90.0%)	AFAT—Factor 2	1.72 (0.48)	1.61 (0.52)	0.28	0.25
	AFAT—Factor 3	2.06 (0.67)	1.93 (0.66)	0.38	0.20
	AFAT—Global score	1.61 (0.38)	1.54 (0.38)	0.40	0.19

* *T-Student's test with normalized data (natural log); Bold values significant differences (p < 0.0125 – Bonferroni's correction)*.

**Table 4:** Dietitians and nutrition students' willingness to recommend each dietitian and Antifat Attitudes Test (AFAT) scale and subscales scores.

	**Would you recommend this dietitian for a friend or family member?**				
**Profiles (% yes answers)**	**Variables**	**Yes**	**No**	p^*^	**d**
Woman with obesity	AFAT—Factor 1	1.19 (0.26)	1.39 (0.39)	**<0.001**	**0.70**
(82.8%)	AFAT—Factor 2	1.70 (0.44)	2.04 (0.51)	**<0.001**	**0.70**
	AFAT—Factor 3	1.84 (0.54)	2.49 (0.60)	**<0.001**	**1.09**
	AFAT—Global score	1.51 (0.31)	1.87 (0.38)	**<0.001**	**1.06**
Woman without obesity	AFAT—Factor 1	1.25 (0.34)	1.50 (0.49)	0.024	0.62
(95.5%)	AFAT—Factor 2	1.76 (0.55)	2.20 (0.56)	**0.005**	**0.77**
	AFAT—Factor 3	1.96 (0.64)	2.16 (0.80)	0.32	0.27
	AFAT—Global score	1.60 (0.42)	1.86 (0.54)	0.02	0.63
Man with obesity	AFAT—Factor 1	1.22 (0.34)	1.38 (0.36)	**<0.001**	**0.53**
(78.1%)	AFAT—Factor 2	1.67 (0.52)	2.20 (0.53)	**<0.001**	**0.92**
	AFAT—Factor 3	1.88 (0.61)	2.30 (0.69)	**<0.001**	**0.64**
	AFAT—Global score	1.53 (0.39)	1.85 (0.44)	**<0.001**	**0.83**
Man without obesity	AFAT—Factor 1	1.21 (0.28)	1.27 (0.36)	0.21	0.17
(81.3%)	AFAT—Factor 2	1.75 (0.47)	1.80 (0.49)	0.44	0.10
	AFAT—Factor 3	1.94 (0.60)	2.01 (0.61)	0.38	0.12
	AFAT—Global score	1.56 (0.34)	1.62 (0.39)	0.27	0.15
Older woman	AFAT—Factor 1	1.23 (0.31)	1.42 (0.46)	0.12	0.44
(94.4%)	AFAT—Factor 2	1.73 (0.49)	2.16 (0.56)	0.30	0.29
	AFAT—Factor 3	1.91 (0.58)	2.15 (0.68)	0.03	0.59
	AFAT—Global score	1.55 (0.36)	1.83 (0.45)	0.07	0.52
Older man	AFAT—Factor 1	1.23 (0.31)	1.50 (0.44)	0.90	0.02
(94.3%)	AFAT—Factor 2	1.77 (0.52)	2.10 (0.65)	0.28	0.25
	AFAT—Factor 3	1.97 (0.63)	2.40 (0.84)	0.38	0.20
	AFAT—Global score	1.60 (0.39)	1.91 (0.54)	0.40	0.19

* *T-Student's test with normalized data (natural log); Bold values significant differences (p < 0.0125 – Bonferroni's correction)*.

Regarding dietitians and nutrition students, the percentage of participants that would recommend the woman with obesity, the man with obesity, and man without obesity was lower than other profiles (Table 4). Those who would not recommend the dietitians with obesity (man and woman) showed higher antifat attitudes in the three subscales and global score, compared to those who would recommend the dietitians, with medium or high effect size (*p* < 0.001, and d ranging from 0.53 to 1.09). Meanwhile, 95.5% would recommend the woman without obesity to a friend or family member. Those who answered “No” showed higher anti-fat attitudes in the subscale 2 (*p* = 0.005, *d* = 0.77). Regarding the other profiles, 81.3% would recommend the man without obesity, and 94.4 and 94.3% would recommend the older woman and the older man, respectively, with no difference between the “Yes” or “No” answers and the AFAT scores.

### Attributes for a Good Dietitian

The most checked attributes for a good dietitian (Table 5) were “communicative, ” “attentive,” “helpful,” “trustworthy,” “innovative, ” “friendly, ” “good-natured, ” and “with experience” with more than 58.9% in all groups. In general, the competence- and warmth-oriented adjectives showed the highest percentages. A significant percentage of dietitians and nutrition students and more than half of the laypeople agreed that a good professional would be an individual within an ideal weight. Meanwhile, the attributes that participants least selected to be related to a good dietitian were “shaven, ” “no make-up, ” “have Facebook^®^, ” “wearing white clothes, ” “fat, ” and “wear social clothes.”

**Table 5:** Adjectives attributed to a good dietitian according to laypeople, registered dietitians, and nutrition students.

**Adjectives group**	**Adjectives**	**Laypeople** **(%)**	**Registered** **dietitians** **(%)**	**Nutrition** **students** **(%)**	***p*** ^*^
Physical appearance	Within the ideal weight	52.1a	30.7b	36.7b	**<0.001**
	Thin	14.4	9.8	13.0	0.17
	Fat	7.1	6.8	9.3	0.43
	Man	38.5a	29.8b	38.3a	**0.02**
	Woman	47.6a	32.1b	41.7ab	**<0.001**
	Young	21.9a	15.5b	23.0a	**0.03**
	Older	17.1	13.1	19.7	0.08
Professional appearance (dress code)	Wear white coat	15.9	16.4	21.0	0.17
	Wear social clothes	6.6	8.3	10.3	0.20
	No make-up	3.3b	4.5ab	8.0a	**0.02**
	With make-up	8.6b	10.4b	15.0a	**0.02**
	Wear white clothes	9.6a	4.2b	7.3ab	**0.02**
	Shaven	2.8	3.3	3.3	0.89
Warmth-oriented	Good-natured	59.4b	59.5b	75.7a	**<0.001**
	Communicative	90.4b	94.0ab	97.7a	**<0.001**
	Friendly	66.5b	68.5ab	81.7a	**<0.001**
	Attentive	89.2	91.4	93.7	0.11
	Helpful	74.8c	87.5b	89.7a	**<0.001**
Competence-oriented	With experience	68.3a	58.9b	60.3b	**0.02**
	Postgraduated	41.1	40.2	37.7	0.65
	Well referenced in the region	57.7a	44.0b	54.7ab	**0.001**
	Have specialization	56.9a	49.1b	54.7ab	**0.02**
	Trustworthy	81.1b	84.8ab	88.9a	**0.02**
Social- and innovativeness-oriented	Have Facebook®	6.3	5.1	5.0	0.69
	Have Instagram®	16.4b	14.9b	22.0a	**0.04**
	Make appointments online	25.2a	17.9b	24.0a	**0.04**
	Make appointments by WhatsApp ®	19.9a	4.8c	7.3b	**<0.001**
	Innovative	61.5c	78.0b	85.7a	**<0.001**

* *Chi-square test;*

*a, b, c—heterogeneous groups at z > 1.96 (standardized residuals). Bold values significant differences (p < 0.05)*.

There were significant differences between the three study groups regarding the attributes related to a good professional. Laypeople showed the highest percentages for: within an ideal weight, have experience and make appointments by WhatsApp®, with a significant difference compared to dietitians and nutrition students. Also, they rated more than dietitians that a good professional should: have a specialization, make appointments online, be well-referenced in the region, wear white clothes and be a man or a woman—but they were not different from nutrition students about this. Nutrition students, in turn, showed the highest percentages for innovative, good-natured, wear make-up, have Instagram®, and helpful—with significant differences compared to dietitians and laypeople. Also, they showed the highest percentage for communicative, do not wear make-up, friendly, and trustworthy—but with a significant difference only from laypeople—and young—with a significant difference only from dietitians.

## Discussion

### General Discussion

This study addressed the position of dietitians with different profiles on the SCM map and their cluster membership according to particular groups (laypeople, dietitians, and nutrition students). This is the first study that applied the SCM to evaluate weight stigma toward professional profiles (registered dietitians). The study is also innovative when using images of different profiles to understand antifat prejudice and obesity discrimination better. The use of images could be beneficial to reduce participant subjectivity when evaluating a specific profile and could promote less salient responses, reducing some possible bias in the SCM map. In general, profiles with obesity were evaluated differently from profiles without obesity. The cluster memberships were different for laypeople compared with dietitians and students. For laypeople, the older profiles and the woman without obesity emerged as a high competence cluster (HC-MW), the woman with obesity as high warmth cluster (MC-HW), and the normal weight and man with obesity as low competence and warmth cluster (LC-LW). Differently, for dietitians and students, no low competence cluster emerged, and an HC-HW cluster (with the woman with obesity and older woman) was observed. These results indicate that the general population perceives dietitians' as less competent because of their high weight. The result is different when a dietitian or student evaluates their peer, especially for women profiles. Finally, the HC-LW cluster did not appear in any group. An HC-LW stereotype combines competence with coldness, generally associated with business positions (e.g., managers), rich persons, and some ethnic stereotypes (e.g., Asians) (3, 37), so this result was expected since we studied health professionals.

The present results confirmed the hypothesis of ambivalent (mixed) stereotypes for dietitians, especially in the laypeople group. The laypeople group attributed greater warmth and lower competence to the woman with obesity but similar rates of higher warmth and competence to the woman without obesity. The two profiles are from the same person, but a heavier bodyweight decreased the belief about her competence. The warmth dimension is generally related to subordination. High-warmth profiles are perceived as low-status and not competitive when associated with lower competence (2). This stigmatizing view corroborates with reports of dietitians with obesity experiencing weight-based discrimination at work (25). In a Brazilian study with dietitians, it is evident that obesity is an obstacle to employment and permanence in workspaces (25). Furthermore, the same study reaffirmed that an individual with a body considered obese by society suffers negative interference in their daily life. In this case, they are seen as a failure, which provokes the need for “policing” themselves and harder efforts to guarantee their permanence at work (25). This pressure is further reinforced in our study. When asked, 52% of laypeople, 31% of dietitians, and 37% of students stated that a good dietitian should be “within an ideal weight.” This biased view of obesity brings harmful consequences. For example, dietitians with obesity have looked to unscientific and ineffective long-term methods to lose weight, such as anorexic drugs, fad diets, meal replacement shakes, and teas (24). O'Brien et al. (28) identified “beliefs of innate superiority” in a college students sample on which according to them, PWO deserve fewer privileges and opportunities than people without obesity. In accordance, other studies with nutrition students have shown that they evaluated PWO more negatively and expressed negative judgments regarding this population's self-care, discipline, and diet quality. Also, they imagined that they would need more rigor and patience to “deal” with PWO in their future practices (38, 39).

As also found in this study, the association of obesity with warmth is common (9), resulting in the “fat-jolly” stereotype. The warmth dimension predicts active facilitation, eliciting feelings of help, tolerance, happiness, and honesty (3). However, when associated with lower competence, the high warmth elicits pity (3). This feeling can make people distrust their competence, harming their health, work, and life in general. The lower competence attributed to the profiles with obesity by laypeople—compared to dietitians and students—may be a response of weight stigma, which has several forms of manifestation, with the preconceived judgment—for example, that these individuals are less competent (40). Weight stigma is widespread and still socially acceptable. This stigma may affect different domains, such as the labor market, the educational system, and even healthcare settings (41–44). On the other hand, in this study, dietitians and nutrition students had a more positive view of profiles with obesity—more specifically the woman with obesity—than laypeople, and the same happened when antifat attitudes were assessed (32). Lower weight stigma in dietitians and nutrition students may be due to receiving education on the causes of obesity. The curriculum of nutrition schools in Brazil addresses the multifactorial etiology of obesity (45). With the increasing interest in the nutritional approach based on the sociocultural and subjective aspects of eating (46), dietitians and nutrition students may see obesity with a broader view and not attribute the causes of obesity to the individual. However, despite efforts to minimize it, the possibility that social desirability bias may have occurred cannot be excluded. When dietitians and nutrition students realized what they were being evaluated on, they may have been more likely to answer in a way they consider most desired and accepted by society, regardless of whether it is true or not (47). Still, considering the results abovementioned with nutrition students (38, 39), it is possible to suggest that the stigmatized views of PWO might perpetuate in their future practice, which should be further investigated.

The man without obesity and the older man were rated as more competent than warm in all groups. This ambivalence did not happen with the man with obesity, who was rated with low average scores in both dimensions. The traditional stereotype of men is ambivalent, and when compared to other groups, men are a less-liked but respected quadrant of a warmth X competence space (2). In this space, perceived competence (i.e., agency) is predicted by perceived social status, both of which favor men. At the same time, perceived lack of warmth (i.e., communality) is predicted by perceived competitiveness. This combination represents successful or dominant groups (e.g., men, rich people, Jewish people, Asian people). Women, on the other hand, are attributed bothnegative traits (e.g., submissive, dependent) and positive traits associated with communality or “other-directed” (48). Women in the traditional female role of housewife are in the warm but not competent quadrant in mapping social groups (2). However, given the context of professional dietitians in Brazil, the result was different from the expected, based on the theory. Women were better evaluated in both dimensions, especially when compared to the man with obesity and the man without obesity. Despite evidence showing men as more competent but not warm, perhaps in predominantly female professions—such as nutrition and dietetics undergraduate degree course in Brazil (49)—women can be seen as more competent (and warm) than men. This result is supported by the current findings that “be a woman” was more cited than “be a man” as a characteristic of a good dietitian. In addition, the samples of dietitians and nutrition students are predominantly female, which may have influenced these results.

Some profiles presented an ambivalent stereotype, such as the woman with obesity, the man without obesity, and the older man. This result was expected since many social stereotypes are ambivalent in diversified cultures (50). However, societies vary in using ambivalent stereotypes; for example, more unequal societies display more ambivalent stereotypes (51). Our study was applied in Brazil, a country with high social inequality (52), which may have predicted the ambivalence of some stereotypes, explaining the maintenance of the current social system. However, our sample showed a high educational level and income—different from the Brazilian population—which may have influenced the unambivalence of the other studied profiles. A cross-sectional study showed that peace-conflict predicts ambivalence (53). Both extremely peaceful and conflictual nations display unambivalent “us vs. them” patterns, whereas intermediate peace-conflict predicts high ambivalence. Also, higher inequality predicts more ambivalent stereotypes clusters. Therefore, inequality and intermediate peace-conflict predict ambivalent stereotypes, explaining complicated intergroup relations and maintaining social system stability (53). This discussion is grounded on how cultures perceive people from different countries or cultures. However, we believe that cultural aspects can also change the perception of professionals, fostering ambivalent stereotypes. The presence of ambivalent stereotypes considering different health professions could be assessed in the future.

When asked if they would consult with each dietitian profiles, laypeople showed more negative responses to the man with obesity, the woman with obesity, and the man without obesity. In addition, those who answered that they would not consult with the dietitians with obesity had more negative attitudes toward obesity, according to the AFAT. Dietitians and nutrition students presented similar results, however, they were questioned if they would recommend that dietitian for a family member or friend. Profiles with obesity and the man without obesity presented higher rejection. This finding suggests that dietitians with obesity may experience different challenges, and be less likely to be consulted and used than dietitians with a normal weight. In this study, negative attitudes were linked with different treatment toward dietitians with obesity (less likely to be consulted or recommended to friends and family). Other surveys have affirmed the rejection and devaluation of PWO in the workplace (22, 43). In the case of dietitians with obesity, their professional performance is challenged and often limited because of their weight (25). This stigma can affect health, as evidenced in studies showing the relationship between weight stigma experiences and deleterious health-related behaviors (e.g., poor diet, decreased motivation for physical activity, physiological stress, worse weight loss outcomes) (16). Furthermore, the cyclic obesity/weight-based stigma model proposes that such stressful experiences of weight can undermine health and lead to further weight gain (54).

Finally, laypeople differed from dietitians and students when attributing qualities for a “good dietitian.” About 50% of laypeople stated that a good dietitian should be a woman and be within an ideal weight. Adjectives related to competence (e.g., with experience, have a specialization, trustworthy) and warmth (e.g., good-natured, friendly, communicative) were also frequently cited (>50%). One adjective of interest is “innovative” The innovative term can refer to non-evidence-based strategies, which arise from time to time and can confuse people. Many of these innovative strategies are seen as effective in weight loss, and healthcare professionals may use these strategies with their patients or clients (55). However, they are generally based on poor-quality studies and could be a risk factor for disordered eating behavior. This perception could be further studied since it can negatively shape dietitians' protocols. Generally, clothes are perceived as a critical driver for health professionals' confidence (56). However, in our sample, little attention was given to this aspect. Perhaps when confronted with a set of characteristics directly linked to dietitians' competence and warmth, the participants downplayed the importance of visual features such as clothing, make-up, and beard.

### Limitations and Further Research

This study has some limitations. Firstly, the data was collected online *via* social media, i.e., the participants had access to the internet and are likely to be readily visible—as they use social media, are computer literate and are likely to have a particular interest in the topic. Moreover, most people in the three samples were young, highly educated, with high family income, and female. Their BMI was generally normal or slightly overweight (laypeople), unlike in the Brazilian population where the percentage of overweight and obesity is currently 55.4 and 20.3%, respectively (57). This female predominance, especially in the groups of dietitians (94.6%) and nutrition students (92.7%), was expected since nutrition is a profession dominated by women in Brazil, as shown in a study by the Brazilian Federal Council of Nutritionists, in which 94.1% of the sample was female (49). This may have affected the results of this study as women seem to hold less weight-based discrimination than men (58). Related, in the SCM evaluation of dietitians and nutrition students is possible to see a preference for the women stereotypes compared to laypeople's evaluation. However, this limitation is difficult to control due to the characteristics of the profession in Brazil.

We could not find any male (pre- and post-bariatric surgery) model fully smiling. The male model (with and without obesity) showed some differences compared to other profiles, and this result could be affected by this aspect (i.e., less attentive and friendly than others). This could pose some bias and could be further investigated in the future. However, this bias could affect only comparisons with different profiles (e.g., women, older men, and women) and not paired comparisons.

Furthermore, individuals responded to profiles with obesity more positively than we expected, especially in the dietitians and nutrition students groups. Even using images to reduce bias, the responses may have been affected by social desirability bias (i.e., an individual's propensity to answer what they consider most appropriate and accepted by society, whether the answer is true or not) (47). Future studies adopting implicit methods or other approaches to minimize social desirability are recommended. Qualitative research methods can be used to better explore and identify the stigmas associated with obesity. In in-depth interviews or focus groups, participants have more freedom to respond and different stimuli can be offered.

## Conclusion

This study provides useful information about the perception of dietitians with obesity. There was some evidence that the woman with obesity was perceived as a less competent dietitian but highly warm. The men profiles were perceived as less competent and warm than women profiles. We also confirmed the hypothesis of ambivalent stereotypes. The woman with obesity was rated as more warm than competent by laypeople and nutrition students, and the man without obesity and the older man were rated as more competent than warm by the three study groups. When compared to the results of dietitians and nutrition students, laypeople perceived the dietitians with obesity as less competent. This is a common characteristic attributed to PWO, and a manifestation of weight stigma. People with higher antifat attitudes also presented negative attitudes toward profiles with obesity.

The weight stigma in healthcare professionals and its negative consequences for the patients' health is well-documented. However, research in the opposite direction—that is, on weight stigma from other similar professionals or laypeople toward professionals with obesity—is scarce. This is the first study comparing attitudes and perceptions of different groups (i.e., peers and laypeople). Our data could foster important discussions about weight stigma and emphasize the need to increase population awareness about obesity and its causes, for example, in undergraduate courses. Dietitians are not “weight loss professionals.” An improved discussion could promote positive changes in the work environment and the well-being of PWO, including health professionals. This, consequently, would support improved patient-professional relationships and patient's long-term outcomes.

## Data Availability Statement

The raw data supporting the conclusions of this article will be made available by the authors, without undue reservation. The detailed results of the SCM variables and the pre-test analysis can be found in the Supplementary Material.

## Ethics Statement

The studies involving human participants were reviewed and approved by the University of Campinas Ethics Committee approved the study (Protocol: 30637020.5.0000.5404). The patients/participants provided their written informed consent to participate in this study.

## Author Contributions

GC: conceptualization, formal analysis, investigation, visualization, and writing—original draft. JC-F and NB: conceptualization, methodology, supervision, writing—review, and editing. MU: visualization, writing—review, and editing. DC: conceptualization, formal analysis, software, methodology, investigation, funding acquisition, supervision, writing—review, and editing. All authors contributed to the article and approved the submitted version.

## Funding

This study was supported by São Paulo Research Foundation (FAPESP) grant #2019/10936-0. This study was partially funded by CAPES—Coordenação de Aperfeiçoamento de Pessoal de Nível Superior (Coordination for the Improvement of Higher Education Personnel), financial code #88887.486890/2020-00 and #001.

## Conflict of Interest

The authors declare that the research was conducted in the absence of any commercial or financial relationships that could be construed as a potential conflict of interest.

## Publisher's Note

All claims expressed in this article are solely those of the authors and do not necessarily represent those of their affiliated organizations, or those of the publisher, the editors and the reviewers. Any product that may be evaluated in this article, or claim that may be made by its manufacturer, is not guaranteed or endorsed by the publisher.
