# Supplementary material for: Are Dietitians With Obesity Perceived as Competent and Warm? Applying the Stereotype Content Model to Weight Stigma in Brazil
Source: Front Nutr. 2022 Feb 25;9:813344. doi: 10.3389/fnut.2022.813344 (PMC8916106; doi:10.3389/fnut.2022.813344)
Supplement: Supplementary file 1 [file Data_Sheet_1.docx]

Supplementary Material

# Supplementary Data

**1.2. Supplementary Tables**

**Supplementary Table 1.** Laypeople’s SCM evaluation

|  | Competence | Dimension |  | Warmth | Dimension | |  |  |
| --- | --- | --- | --- | --- | --- | --- | --- | --- |
| Stereotype | Competent | Confident | Intelligent | Warm | Sincere | Good-natured | | Probability of following advice |
| Man with obesity | 3.02c; 0.94 | 3.02c; 0.93 | 3.45b;0.84 | 3.20c;0.99 | 3.40c;0.86 | 3.19c;0.99 | | 3.53c;0.98 |
| Man without obesity | 3.42b; 0.72 | 3.30bc; 0.77 | 3.59b;0.75 | 2.68d;0.82 | 3.48c;0.86 | 2.82d;0.85 | | 3.74bc;0.84 |
| Woman with obesity | 3.26bc; 0.98 | 3.38b; 0.94 | 3.90a;0.80 | 4.23a;0.76 | 3.77ab;0.88 | 4.06a;0.84 | | 3.81abc;1.03 |
| Woman without obesity | 3.73a; 0.78 | 3.69a; 0.73 | 3.86a;0.72 | 3.71b;0.82 | 3.63bc;0.74 | 3.56b;0.89 | | 4.02a;0.72 |
| Older man | 3.90a; 0.83 | 3.80a; 0.77 | 4.03a;0.75 | 3.25c;0.87 | 3.89ab;0.79 | 3.24c;0.87 | | 3.97ab;0.83 |
| Older woman | 3.85a; 0.81 | 3.81a; 0.75 | 3.94a;0.69 | 3.86b;0.81 | 3.89a;0.81 | 3.79ab;0.79 | | 4.07a;0.75 |

Letters on the same column indicates significant difference Bonferroni’s test (p<0.05).

**Supplementary Table 2.** Registered dietitians’ SCM evaluation

|  | Competence | | Dimension | |  | | Warmth | | Dimension | |  | |
| --- | --- | --- | --- | --- | --- | --- | --- | --- | --- | --- | --- | --- |
| Stereotype | | Competent* | | Confident | | Intelligent | | Warmth | | Sincere | | Good-natured |
| Man with obesity | | 3.73d;0.87 | | 3.68c;0.86 | | 3.96b;0.80 | | 3.51c;1.01 | | 3.83ab;0.85 | | 3.45cd;0.98 |
| Man without obesity | | 3.76cd;0.83 | | 3.70bc;0.88 | | 3.88b;0.86 | | 3.09d;1.07 | | 3.72b;1.00 | | 3.11d;1.02 |
| Woman with obesity | | 4.06abc;0.85 | | 4.05a.0.85 | | 4.31a;0.68 | | 4.43a;0.62 | | 4.13a;0.76 | | 4.27a;0.74 |
| Woman without obesity | | 4.01bcd;0.75 | | 3.99ab;0.74 | | 4.01b;0.74 | | 4.00b;0.79 | | 3.76b;0.82 | | 3.87b;0.83 |
| Older man | | 4.32a;0.73 | | 4.15a;0.82 | | 4.28a;0.72 | | 3.59c;0.97 | | 4.09a;0.78 | | 3.51c;0.97 |
| Older woman | | 4.19ab;0.68 | | 4.16a;0.66 | | 4.15ab.0.67 | | 4.11b;0.76 | | 4.10a;0.76 | | 4.07ab;0.78 |

Letters on the same column indicates significant difference Bonferroni’s test (p<0.05).

**Supplementary Table 3.** Nutrition students’ SCM evaluation

|  | Competence | Dimension |  | Warmth | Dimension |  |
| --- | --- | --- | --- | --- | --- | --- |
| Stereotype | Competent | Confident | Intelligent | Warmth | Sincere | Good-natured |
| Man with obesity | 3.60c;0.94 | 3.65b;0.92 | 3.99bc;0.72 | 3.26c;1.08 | 3.85ab;0.86 | 3.36cd;1.09 |
| Man without obesity | 3.75c;0.83 | 3.63b;0.88 | 3.82c.0.75 | 2.83d;1.00 | 3.83ab;0.85 | 2.98d;1.04 |
| Woman with obesity | 3.80bc;0.98 | 3.87ab;0.98 | 4.21ab;0.72 | 4.49a;0.64 | 4.13a;0.84 | 4.35a;0.69 |
| Woman without obesity | 4.07ab;0.75 | 4.08a;0.76 | 4.13ab;0.69 | 4.05b;0.81 | 3.79b;0.81 | 3.89b;0.84 |
| Older man | 4.25a;0.68 | 4.10a;0.74 | 4.31a;0.60 | 3.42c;0.92 | 3.97ab;0.79 | 3.50c;0.86 |
| Older woman | 4.14ab;0.82 | 4.06a;0.83 | 4.16ab;0.79 | 4.11b;0.81 | 4.01ab;0.84 | 4.11ab;0.79 |

Letters on the same column indicates significant difference Bonferroni’s test (p<0.05).

**Supplementary Table 4.** Composite reliability of Competence and Warmth dimensions

| Variables | Dimensions | | Composite Reliability | | Average Variance Extracted | |
| --- | --- | --- | --- | --- | --- | --- |
| Woman with obesity | Competence | 0.93 | | 0.81 | |  |
|  | Warmth | 0.74 | | 0.51 | |  |
| Woman without obesity | Competence | 0.93 | | 0.81 | |  |
|  | Warmth | 0.85 | | 0.66 | |  |
| Man without obesity | Competence | 0.86 | | 0.68 | |  |
|  | Warmth | 0.95 | | 0.86 | |  |
| Man with obesity | Competence | 0.88 | | 0.70 | |  |
|  | Warmth | 0.94 | | 0.84 | |  |
| Older woman | Competence | 0.86 | | 0.67 | |  |
|  | Warmth | 0.94 | | 0.83 | |  |
| Older man | Competence | 0.87 | | 0.69 | |  |
|  | Warmth | 0.93 | | 0.82 | |  |

**Supplementary Table 5.** Ambivalence of stereotypes. RD: registered dietitians. M_diff_: Mean Difference.

| Group | Stereotypes | M_diff_ | *p** | d |
| --- | --- | --- | --- | --- |
|  | Woman with obesity | 0.50 | <0.001 | 0.71 |
|  | Woman without obesity | 0.12 | 0.001 | 0.23 |
| Laypeople | Man without obesity | 0.44 | <0.001 | 0.73 |
|  | Man with obesity | 0.09 | 0.04 | 0.14 |
|  | Older woman | 0.01 | 0.72 | 0.02 |
|  | Older man | 0.46 | <0.001 | 0.72 |
|  | Woman with obesity | 0.13 | 0.002 | 0.23 |
|  | Woman without obesity | 0.12 | 0.005 | 0.22 |
| RD | Man without obesity | 0.47 | <0.001 | 0.75 |
|  | Man with obesity | 0.19 | 0.001 | 0.27 |
|  | Older woman | 0.05 | 0.19 | 0.10 |
|  | Older man | 0.50 | <0.001 | 0.79 |
|  | Woman with obesity | 0.36 | <0.001 | 0.52 |
|  | Woman without obesity | 0.18 | <0.001 | 0.31 |
| Nutrition students | Man without obesity | 0.52 | <0.001 | 0.85 |
|  | Man with obesity | 0.25 | <0.001 | 0.36 |
|  | Older woman | 0.06 | 0.14 | 0.12 |
|  | Older man | 0.60 | <0.001 | 0.93 |

* Paired t-Student’s test

**Supplementary Table 6.** Validation process of the different profiles (n= 61).

| **Image** | **Image identification** | **Gender indication** | | **Assigned weight (kg) (Mean;SD)** | | **Assigned age (years) (Mean;SD)** | |
| --- | --- | --- | --- | --- | --- | --- | --- |
| 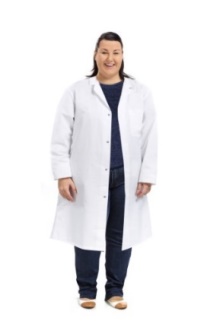 | Woman with obesity | | 100% woman | | 91.4; 14.3 | | 31.40; 5.38 |
| 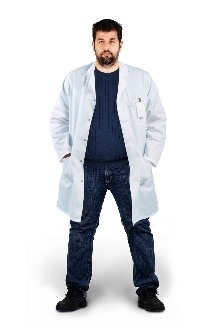 | Man with obesity | | 100% man | 99.8; 12.1 | | 35.85; 5.62 | |
| 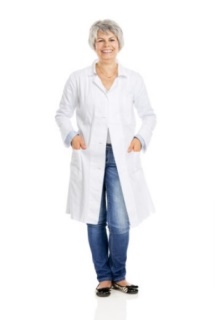 | Older woman | | 100% woman | 63.8; 5.9 | | 55.32; 6.38 | |
| 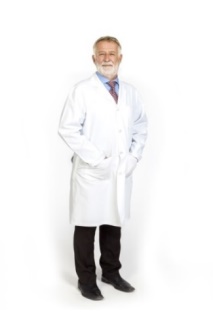 | Older man | | 100% man | 78.5; 6.0 | | 63.90; 5.98 | |
| 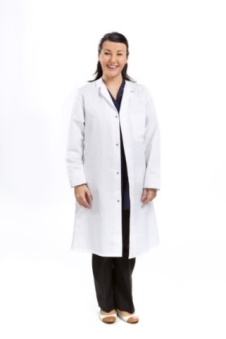 | Woman without obesity | | 100% woman | 66.1; 5.9 | | 34.59; 6.90 | |
| 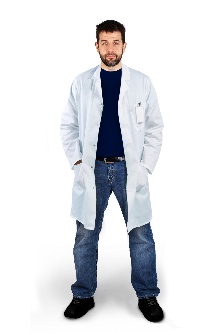 | Man without obesity | | 100% man | 79.2; 6.4 | | 34.14; 6.14 | |
